# Extracorporeal Cytokine Removal in Critically Ill COVID-19 Patients: A Case Series

**DOI:** 10.3389/fmed.2021.760435

**Published:** 2021-11-19

**Authors:** Marcell Virág, Máté Rottler, Klementina Ocskay, Tamás Leiner, Balázs Horváth, Daniel Adam Blanco, Alexa Vasquez, László Bucsi, Ágnes Sárkány, Zsolt Molnár

**Affiliations:** ^1^Medical School, Institute for Translational Medicine, University of Pécs, Pécs, Hungary; ^2^Department of Anesthesiology and Intensive Therapy, Szent György University Teaching Hospital of Fejér County, Székesfehérvár, Hungary; ^3^Doctoral School of Clinical Medicine, University of Szeged, Szeged, Hungary; ^4^Anaesthetic Department, Hinchingbrooke Hospital, North West Anglia National Health Service (NHS) Foundation Trust, Huntingdon, United Kingdom; ^5^CytoSorbents Europe, Berlin, Germany; ^6^Szent György University Teaching Hospital of Fejér County, Székesfehérvár, Hungary; ^7^Department of Anesthesiology and Intensive Therapy, Poznan University of Medical Sciences, Poznan, Poland; ^8^Department of Anesthesiology and Intensive Therapy, Semmelweis University, Budapest, Hungary

**Keywords:** ARDS, cytokine storm, CRRT, COVID-19, hemoadsorption, Cytosorb

## Abstract

**Introduction:** Extracorporeal hemoadsorption (HA) is a potential adjunctive therapy in severe cases of COVID-19 associated pneumonia. In this retrospective study we report data from critically ill patients treated with HA during the first and second wave of the pandemic.

**Patients and Methods:** All patients, who received HA therapy with CytoSorb within the first 96 h of intensive care unit (ICU) admission without hospital-acquired bacterial superinfection, were included. Clinical and laboratory data were collected: on admission, before (T_B_) and after (T_A_) HA therapy.

**Results:** Out of the 367 COVID-19 cases, 13 patients were treated with CytoSorb, also requiring mechanical ventilation and renal replacement therapy. All patients were alive at the end of HA, but only 3 survived hospital stay. From T_B_-T_A_ there was a tendency of decreasing norepinephrine requirement: 193.7 [IQR: 34.8–270.4] to 50.2 [6.5–243.5] ug/kg/day and increasing PaO2/FiO2 ratio 127.8 (95% CI: 96.0–159.6) to 155.0 (115.3–194.6) mmHg but they did not reach statistical significance (*p* = 0.14 and 0.58, respectively). Treatment related adverse events were not reported.

**Conclusion:** The treatment was well-tolerated, and there was a tendency toward an improvement in vasopressor need and oxygenation during the course of HA. These observations render the need for prospective randomized trials.

## Introduction

Treating critically ill Coronavirus Disease 19 (COVID-19) patients has become the most significant challenge intensive care has ever faced. The vast number of patients requiring care simultaneously, and the novelty and unpredictability of the virus created an unprecedented environment for all involved. Hospitals were overwhelmed by the continuous influx of severely unwell patients, and intensive care units (ICUs) expanded beyond their original footprint. Critical care mortality of COVID-19 patients significantly exceeded the mortality of any other viral pneumonia (37.9 vs. 22.0%) (1).

Due to the early introduction of lockdown during the first wave of the pandemic, Hungary experienced significantly less pressure on its healthcare system compared to most of the Western European countries. However, it was hit very hard by the next consecutive waves during the autumn and winter of 2020–2021, which resulted in one of the, if not the highest rates of COVID-19 mortality per capita in the world (304.33 deaths/100,000) (2).

Early reports of observations indicated that cytokines might play a considerable role in the development of severe COVID-19. Patients who required critical care admission had higher levels of cytokines. Furthermore, tumour necrosis factor-alfa (TNFa) levels correlated with disease severity, and high interleukin 6 (IL-6), C-reactive protein (CRP), D-Dimer and ferritin levels were found to be predictors of mortality (3–5).

There is some evidence that with extracorporeal cytokine adsorption, substantial IL-6 removal is achievable in severely ill patients with septic shock, acute respiratory distress syndrome (ARDS), and multi-organ failure (6). In a Hungarian proof of concept randomised, controlled pilot study, a significant reduction in vasopressor need and serum pro-calcitonin levels were found (7). Moreover, in a retrospective study of patients with septic shock, cytokine removal was associated with a decreased observed vs. expected 28-day all-cause mortality (8).

In our tertiary intensive care unit (ICU) we have also used hemoadsorption (HA) as an adjuvant therapy in selected patients with septic shock since 2016. During the COVID-19 pandemic our institute served as one of the biggest referral centres in the country, with 120 dedicated COVID-19 ICU beds during the peak of the pandemic (un-published data). As the situation proved desperate, we also reached out for adjunctive therapies such as cytokine adsorption in the most severely ill COVID-19 patients.

The aim of the current retrospective study is to summarise our experience with extracorporeal cytokine adsorption in critically ill COVID-19 patients admitted to our ICU during the first and second wave of the pandemic.

## Patients and Methods

For this retrospective case-series we screened the records of all patients admitted to the ICU of the Department of Anaesthesia and Intensive Care Medicine at Fejér County St. György University Teaching Hospital, Székesfehérvár, Hungary, between March 1st 2020 and January 31st, 2021. Approval from the local ethics committee was obtained (No: 18/2021.05.11.). Due to the retrospective and anonymized data collection process, patients' informed consent was not deemed necessary.

We identified patients, who had severe, life-threatening COVID-19 confirmed with either polymerase chain reaction (PCR) or rapid antigen testing.

All patients had severe respiratory failure requiring mechanical ventilation and received standard intensive monitoring and therapy based on international guidelines and recommendations.

All patients, who received HA therapy within the first 96 h of ICU admission were considered eligible for the analysis. Hemadsorption treatment was applied as an additional adsorbent cartridge (CytoSorb/CytoSorbents Europe GmbH, Berlin, Germany) integrated into a continuous renal replacement (CRRT) circuit. Patients who received HA more, than up to a maximum of 96 h after ICU admission were presumed to have septic shock due to hospital acquired secondary infections, hence they were excluded. Those, who died within 48 h of the application of the first HA were excluded.

The indication to start CRRT was based on our local protocol: CRRT was commenced in patients with acute renal injury (AKI) stage II according to Kidney Disease Improving Global Outcomes (KDIGO) criteria or severe refractory fluid overload, furthermore in patients without renal indication in order to facilitate HA therapy in hemoperfusion mode only (9).

Executing CRRT with or without HA was at the discretion of the attending senior intensive care physician. Nevertheless, by-and-large the following indications were taken into account: suspicion of hyper-inflammatory state based on elevated inflammatory markers such as CRP and granulocyte count combined with considerable hemodynamic instability necessitating increasing doses of catecholamines and/or severe ARDS defined by the Berlin definition and/or multiple organ failure (10).

Integration of the adsorber into the CRRT circuit (Prismaflex System/Baxter International Inc., Deerfield, IL, USA) was handled by trained intensive care physicians following the manufacturer's instructions. The cartridge was placed in pre-dialyzer position. CRRT was performed in continuous hemodiafiltration mode (CVVHDF), as our standard practise, at a blood flow rate of 100–250 ml/min with systemic unfractionated heparin or regional citrate anticoagulant as recommended by the KDIGO 2012 recommendations (11). Based on the attending physician's decision the cartridges were changed every 12 or 24 h. According to our standard operating procedures, hemoadsorption was discontinued in cases of clinical improvement as indicated by a reduction in the required catecholamine dose, increase in the PaO2/FiO2 ratio, or due to deterioration in the patient's overall condition or no improvement after 2 treatment sessions.

Data collection was undertaken retrospectively by review of our electronic medical records. We gathered pertinent information on demographic data and past medical his-tory. For risk stratification the 4C and sequential organ failure assessment scores (SOFA) were calculated. Relevant clinical data were assessed at three different time points: (1) on admission, (2) at the onset of the adsorbent therapy (“before,” TB), and 3) after the completion of adsorbent therapy (“after,” TA). We measured inflammatory markers such as leukocyte and granulocyte count, CRP, procalcitonin (PCT), hemodynamic parameters such as catecholamine requirement, serum lactate levels; respiratory function as indicated by the PaO2/FiO2 ratio, PaCO2, and renal function assessed by blood urea nitrogen, creatinine and glomerular filtration rate. We analysed the association between the use of HA and changes in SOFA-score, in the PaO2/FiO2 ratio and in catecholamine dose, as well as days on mechanical ventilation, ICU length-of-stay and 28-day mortality.

### Statistical Analysis

Data were collected in a preformatted, anonymized table, which had been used exclusively for all further analyses. All calculations were undertaken by means of descriptive statistics. Continuous variables were expressed as mean (confidence interval, CI) or median [interquartile range, IQR], as appropriate. Statistical analyses and graphs were performed with STATA 15 software using parametric and non-parametric methods for mean and median comparisons as appropriate.

## Results

### Baseline Characteristics

Out of the 367 patients treated with COVID-19 in the ICU, invasive ventilation was necessary in 153 cases. Thirty-Seven patients were on CRRT, and 13 patients were included the current case series suffering from COVID-19 viral pneumonia, who received hemoadsorption therapy with CytoSorb. Three patients were admitted from a COVID-19 medical ward and one patient was transferred from another hospital's ICU. All four patients were admitted to our ICU within 24 h after the onset of symptoms, hence hospital acquired infection was highly unlikely.

Baseline characteristics, comorbidities and on-admission laboratory values of the included patients are presented in Table 1. All patients but one were male, with a mean age of 57 (±11) years. According to the 4C prognostic mortality score most patients (61.6%) had at high or very high risk on admission. Nine patients (69.2%) suffered from some sort of comorbidity, including hypertension, diabetes, or acute myocardial infarction. Regarding on-admission laboratory parameters, as indicated by the average values, granulocyte/lymphocyte ratio, CRP, lactate dehydrogenase, creatinine kinase, glutamic oxaloacetic transaminase, and gamma-glutamyl transferase, were elevated. The applied antiviral and adjunctive therapies are detailed in Table 1.

**Table 1:** Baseline characteristics.

	**Patients (*n* = 13)**
Age, years	62 [48–67]
Male	12 (92.3%)
Charlson comorbidity index	2.77 (1.1–4.4)
4C Mortality score for COVID-19	10.4 (7.6–13.2)
Low risk	1 (7.7%)
Intermediate risk	4 (30.8%)
High risk	5 (38.5%)
Very high risk	3 (23.1%)
Onset of symptoms before admission, days	5.8 (3.8–7.8)
Confirmed by COVID-19 antigen test	3 (23.1%)
Confirmed by PCR	10 (76.9%)
**Comorbidities**	
Hypertension	9 (69.2%)
Diabetes	6 (46.2%)
Gastroesophageal reflux disease	1 (7.7%)
Acute myocardial infarction	2 (15.4%)
Benign prostatic hyperplasia	2 (15.4%)
Other	6 (46.2%)
No comorbidities	4 (30.8%)
**Laboratory values on admission**	
Leukocytes, ×10^3^/μL	8.9 (6.6–11.2)
Lymphocytes, ×10^3^/μL	0.7 [0.58–1.04]
Granulocytes, ×10^3^/μL	7.4 (5.3–9.6)
Granulocyte/lymphocyte ratio	10.0 (6.9–13.1)^*^
C-reactive protein, mg/L	203 (116–290)^*^
Glutamic oxaloacetic transaminase, U/L	96 [42–113]^*^
Glutamate pyruvate transaminase, U/L	75 (40–109)^*^
Gamma-glutamyl transferase, U/L	131.1 (66.3–195.9)^*^
Alkaline phosphatase, U/L	100.1 (68.3–131.9)
Lactate dehydrogenase, U/L	1,164.8 (780.9–1,548.8)^*^
Blood urea nitrogen, mmol/L	6.4 [4.8–13.3]
Creatine kinase, U/L	160 [126–489]^*^
**Initial antiviral therapy**	**11 (84.6%)**
Oseltamivir	4 (30.8%)
Favipiravir	6 (46.2%)
Lopinavir	1 (7.7%)
**Adjunctive therapy**	
Dexamethasone	2 (15.4%)
Hydrocortisone	4 (30.8%)
Methyl-prednisolone	4 (30.8%)
Tocilizumab	4 (30.8%)
Anti-COVID-19 convalescent plasma	5 (38.5%)

*Data are presented as mean (95% CI), median [IQR], or n (%), unless otherwise specified. PCR, polymerase chain reaction. U/L, unit/litre; ICU, intensive care unit*.

* *represents elevated values*.

The mean time between the onset of symptoms and admission to the Emergency Department was 5.9 (95% CI: 4.11–7.55) days. The diagnosis of SARS-CoV2 infection was confirmed by PCR in ten and antigen testing in three cases. Two patients had chest computer tomography and eleven X-Ray, and except for one patient, bilateral pathology was observed. Nine patients were admitted from the Emergency Department to the ICU on the day of admission, three were transferred from the department of COVID-Internal Medicine and one from the ICU of another town.

### Treatment Characteristics

In all cases, hemoadsorption was combined with CRRT. On average 1.6 days elapsed from admission to the application of the first adsorber. Regarding the number of treatments, 1 patient received only one treatment, 5 received two, 6 received three treatments and in 1 case hemoadsorption was repeated for four consecutive sessions.

**Figure 1:**
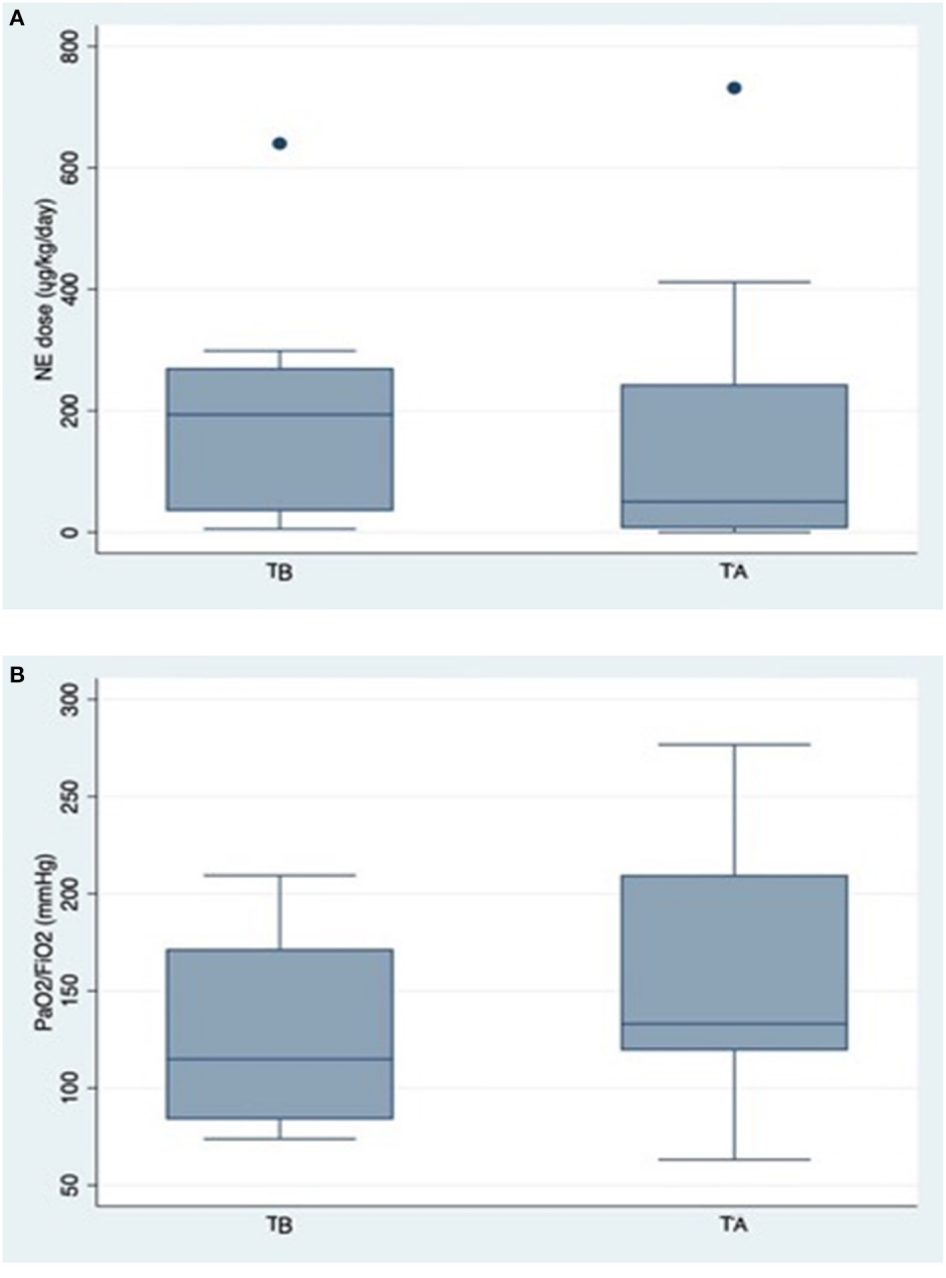
**(A)** Change in norepinephrine requirement during hemoadsorption. NE, Norepinephrine; TB, data collected before the start of hemoadsorption; TA, data collected after the completion of hemoadsorption. Data are presented as box-plots. For statistical analysis Stata 15.1 was used. For explanation see main text. **(B)** Change in the PaO2/FiO2 ratio during hemoadsorption. TB, data collected before the start of hemoadsorption; TA, data collected after the completion of hemoadsorption. Data are presented as box-plots. For statistical analysis Stata 15.1 was used. For explanation see main text.

All patients required mechanical ventilation due to acute hypoxemic respiratory failure. Vasopressor was also required in all patients. In the first 24 h the total dose of norepinephrine was 193.7 ug/kg/day [34.8–270.4] which decreased to 50.2 [6.5–243.5] ug/kg/day, by the end of the course of hemoadsorption, but did not reach statistical significance (Figure 1A). There was a similar tendency of an improvement in the PaO2/FiO2 ratio during the course of hemoadsorption from 127.8 (96.0–159.6) to 155.0 (115.3–194.6) mmHg (Figure 1B). Apart from the leukocyte count, all other recorded laboratory parameters and the SOFA score showed statistically non-significant changes from before to after the therapy (Table 2).

**Table 2:** Before and after comparisons for all patients.

**Laboratory values**	**T_**B**_**	**T_**A**_**	***p*-value**
Leukocytes, ×10^3^/μL	8.5 (6.5–10.4)	13.0 (9.0–16.9)	>0.01
Haemoglobin, g/L	119 (108–129)	115 (105–126)	0.33
Platelets, ×10^3^/μL	190 (140–241)	178 (130–225)	0.52
Creatinine, umol/L	109 [86–264]	104 [67–177]	0.05
Blood urea nitrogen, mmol/L	11.2 (6.9–15.6)	8.4 (5.7–11.2)	0.08
Glomerular filtration rate, mL/min/1.73 m^2^	55.3 (34.1–76.5)	57.5 (38.4–76.6)	0.58
C-reactive protein, mg/L	266.1 (142.0–390.2)	206.4 (129.0–283.8)	0.28
Procalcitonin, ng/mL (*n* = 5)	0.64 [0.20–2.85]	0.49 [0.49–0.62]	0.59
Bilirubin, mmol/L (*n* = 3)	11.0 [10.0–333.0]	106.0 [30.0–182.0]	0.32
**Arterial blood gases**			
pH	7.35 (7.33–7.39)	7.31 (7.23–7.38)	0.28
PaO2, mm Hg	109 (82–135)	99 (85–112)	0.50
PaCO2, mm Hg	43 (37–48)	46 (37–55)	0.47
Plasma bicarbonate, mmol/L	23.3 (20.7–25.8)	22.6 (19.7–25.5)	0.65
Arterial lactate, mmol/L	1.68 [1.23–2.27]	1.58 [1.33–2.25]	0.74
SOFA score	15.0 [15.0–17.5]	14.5 [14.0–16.5]	0.03

*Data are expressed as mean (95% CI) or median [IQR], unless otherwise specified. HA, hemoadsorption. n = number of patients when <13. PaO2, Partial pressure of oxygen. PaCO2, Partial pressure of carbon dioxide. PaO2/FiO2, Ratio of the partial pressure of oxygen and the fraction of inspired oxygen. SOFA, Sequential organ failure assessment*.

Fluid management and inotropic support are summarised in Table 3. The only significant change was a negative fluid balance (TB vs. TA) on the completion of hemoadsorption compared to the onset of implication of the first HA.

**Table 3:** Summary of daily changes in fluid balance and inotropic support requirement.

**Fluid balance**	**T_**B**_**	**T_**A**_**	***p*-value**
Intake fluids per orally, ml	335 (141–529)	554 (150–958)	0.24
Intake fluids intravenously, ml	3,760 (3143–4377)	3,386 (2875–3897)	0.31
Spontaneous urinary output, ml	1,600 [750–2,300]	1,200 [0–3,850]	0.32
Other fluid loss, ml	200 [0–400]	0 [0–200]	0.24
Remove by continuous renal replacement, ml	0 [0–300]	953 [300–2,400]	0.20
Daily fluid balance, ml	1,947 (1,053–2,840)	−322 (−1,388–745)	>0.01
**Vasopressor requirement**			
Norepinephrine, μg/kg/day	193.7 [34.8–270.4]	50.2 [6.5–243.5]	0.48
Dobutamine, μg/kg/day (*n* = 4)	0.0 [0.0–1,005.7]	0.0 [0.0–5,437.5]	0.03

*Data are expressed as mean (95% CI) or median [IQR], unless otherwise specified. HA, hemoadsorption*.

### Overall Outcomes

All patients survived the course of hemoadsorption and were still alive 72 h after initiation of the treatment, hence therapy was not terminated due to deterioration and also no adverse events were detected. Four patients survived 28-days, but overall, only 3 patients (23.1%) were discharged from the hospital alive. ICU length of stay was 14 [5–30] days while patients spent a median 17 [6–30] days in the hospital in total.

## Discussion

To the best of our knowledge, this is the first and largest comprehensive case series on COVID-19 patients treated with hemoadsorption from Eastern Europe—i.e.: from the former socialist block. Although the results showed some improvement in both haemodynamics and oxygenation, likely due to small sample size statistical significance was not achieved. Nevertheless, all patients were alive when treatment was decided to be terminated by the attending medical team, and treatment-related adverse events were not re-ported. Death occurred due to multiple organ failure at least 2 weeks later in most cases, suggesting that hemoadsorption started within 96 h after admission to ICU was well-tolerated and helped to stabilise and overcome the initial critical phase.

The purpose of hemoadsorption in the critically ill in general is to remove excessive inflammatory mediators and by doing so, to attenuate the host immune response. Whether a dysregulated immune response is present or not in COVID-19 patients at all, hence whether hemoadsorption is indicated or not, remains a controversial topic despite extensive publications in the field over the last year. There are several reports indicating elevated cytokine levels in the critically ill forms of COVID-19, some even observed the presence of cytokine storm (12, 13). However, there are also reports contradicting this hypothesis (14, 15). Nevertheless, as the clinical phenotype of COVID-19 is highly variable, ranging from asymptomatic cases to multi organ failure, one would expect a similar variability for the immune response (16). In recent correspondence by Rieder et al. on 8 patients with severe COVID-19 requiring veno-venous-extracorporeal membrane oxygenation (the CYCOV study), patients who received extracorporeal cytokine adsorption had lower IL-6 levels after 72 h of treatment compared to patients without cytokine adsorption (17). However, after completion of the study the difference was not significant (18).

In our patients there are some signals of increased inflammatory activity as indicated by the elevated granulocyte/lymphocyte ratio, and CRP and PCT levels, but these are certainly less pronounced as seen in septic shock or severe ARDS (14). Regardless of the measured inflammatory biomarker levels, the clinical picture of hemodynamic instability requiring vasopressor support, severe ARDS and renal failure indicates the presence of a dysregulated immune response of some sort. Therefore, which biomarkers are the most appropriate to be measured in COVID-19 patients remains to be elucidated (19).

Regarding the clinical effects of hemoadsorption with CytoSorb, there are several, consistent reports of hemodynamic stabilisation before and after the therapy from several fields of critical care including cardiac surgery, septic shock and also recent reports in COVID-19 patients (7, 20, 21). However, the amount of published data on hemoadsorption in COVID-19 patients remains very limited. The largest patient cohort on COVID-19 patients treated with hemoadsorption to date, was published by Alharthy et al. (22). Fifty patients with life threatening COVID-19 and acute kidney injury were treated with CytoSorb. Before and after treatment laboratory and clinical values were compared in the 35 survivors to 15 non-survivors. Most organ functions, including vasopressor need, PaO2/FiO2, and inflammatory biomarker levels improved significantly amongst survivors, while al-most all of these deteriorated in non-survivors. Due to the limited number of patients we could not compare the 10 non-survivors to the 3 survivors in our study. Nevertheless, the tendency was also similar in our cases, although the improvement did not reach statistical significance. This can be explained on the one hand by the small sample size, on the other hand there is some data suggesting the starting HA within 24 h as compared to the average 1.6 days found in our study, could have more profound effects (23).

Our patient cohort is similar to that presented in previous studies, as far as age, comorbidities and treatment modalities are concerned. However, the observed mortality was substantially higher (77%) than in other studies (22, 24, 25), apart from one recent randomised trial in patients requiring extracorporeal membrane oxygenation (ECMO) due to COVID-19 caused respiratory failure (18). In this study, Supady et al. (18) report a striking difference in 28-day survival of 18% in the cytokine adsorption treated vs. 76% in the controls. However, this was a small (17 vs. 17 patients) single centre study. There were also some baseline differences between the groups as far as baseline norepinephrine requirement, D-Dimer levels, and fluid balance were concerned, hence firm conclusions from this study cannot be drawn at present. Finally, this trial included patients on ECMO, which is different from our patient cohort. One most note, that the average 4C mortality score was 10.4, which indicates high risk and corresponds to 31.4–34.9% in-hospital mortality. Five patients were in the high risk and three in the very high risk category. However, these patients all required mechanical ventilation, therefore were of a selected subpopulation with even more severe conditions (26).

However, an important fact is that mortality rate during the 2nd and especially during the 3rd wave of COVID-19 was a lot higher in Eastern Europe in general than in West-ern Europe or in the United States (27–30). Unfortunately, we do not have detailed data on ICU/mechanical ventilation specific outcomes, hence we cannot give a clear explanation either for the differences between East and West Europe nor for the high mortality observed in our current case series.

However, it has long been reported by key opinion leaders of intensive care medicine in the region, that although we are more than 3 decades after the changing of the socialist system, several fundamental issues are still to be solved (31, 32). The most burning is the lack of human resources, including ICU nurses and doctors alike. Although our study did not focus on COVID-19 mortality in general, we cannot exclude that in the overall high ICU mortality, the low number of specialised ICU nurses and doctors and other logistic and organisational factors played a role. Unfortunately, we cannot refer to published data, but during the pandemic in our ICU, the specialised patient-to-nurse ratio often increased above 4:1. This is of utmost importance, as it has been shown by several studies that nurse/patient ratio has an exponential effect on mortality (33–35). In fact, if the patient-to-nurse ratio increases from 1:1 to >2.5:1 per shift, the adjusted risk ratio “shifts, with at least one death” increases from 1 to 3.5 (95% CI: 1.3–9.1) (31). In this region of Europe, we must take this opportunity to learn from the lessons of the pandemic that state-of-the art equipment and treatment strategies, such as CytoSorb, cannot make a difference if the human resources do not match the international/European standards.

### Limitations

Our study has many more limitations than strengths. It is a retrospective, single centre, small sample size case series without a control group. However, there are very few studies in general available on this topic. Furthermore, our results are not negative as far as the treatment's immediate effects are concerned. Regarding long term outcomes, in comparison to international results, ours cannot be compared for the reasons detailed above. We could not present data on IL-6, and several other parameters.

### Conclusions

In this small, singe centre cohort we present the results of COVID-19 patients treated with hemoadsorption during their life-threatening critical illness. The treatment was well-tolerated and there was a tendency toward improvement in both vasopressor need and oxygenation during the course of hemoadsorption. These observations render the need for prospective randomised trials.

## Data Availability Statement

The original contributions presented in the study are included in the article/supplementary material, further inquiries can be directed to the corresponding author/s.

## Ethics Statement

The studies involving human participants were reviewed and approved by Ethics Committee of Fejér County St. György University Teaching Hospital, Székesfehérvár, Hungary (No: 18/2021.05.11). Written informed consent for participation was not required for this study in accordance with the national legislation and the institutional requirements. Written informed consent was not obtained from the individual(s) for the publication of any potentially identifiable images or data included in this article.

## Author Contributions

ZM: conceptualisation, supervision, project administration, and funding acquisition. MR and MV: methodology. MV and BH: software. KO and MR: validation. AV and DB: formal analysis. MV: investigation, resources, and data curation. TL, MR, MV, and KO: writing—original draft preparation. ZM, LB, and ÁS: writing—review and editing. BH: visualisation. All authors have read and agreed to the published version of the manuscript.

## Funding

The article was funded by the project titled GINOP-2.3.2-15-2016-00048–STAY ALIVE is co-financed by the European Union (European Regional Development Fund) within the frame-work of Program Széchenyi 2020, Human Resources Development Operational Program Grant, Grant No. EFOP-3.6.2-16-2017-00006—LIVE LONGER is co-financed by the European Union (European Regional Development Fund) within the framework of Program Széchenyi 2020 and Hungarian National Research, Development and Innovation Office (Grant No. K 138816).

## Conflict of Interest

ZM has received lecture honoraria form PULSION Medical, Germany (member of the Getinge Group), ThermoFisher Scientific and Biotest, Germany and he is a senior medical director at CytoSorbents Europe, Berlin, Germany. DB is employed by CytoSorbents Europe, Berlin, Germany; AV holds a contract as an external consultant with CytoSorbents Europe, Berlin, Germany. The remaining authors declare that the research was conducted in the absence of any commercial or financial relationships that could be construed as a potential conflict of interest.

## Publisher's Note

All claims expressed in this article are solely those of the authors and do not necessarily represent those of their affiliated organizations, or those of the publisher, the editors and the reviewers. Any product that may be evaluated in this article, or claim that may be made by its manufacturer, is not guaranteed or endorsed by the publisher.
